# Misclassification of stunting, underweight and wasting in children 0–5 years of South Asian and Dutch descent: ethnic-specific *v*. WHO criteria

**DOI:** 10.1017/S1368980019004464

**Published:** 2020-08

**Authors:** JA de Wilde, M Peters-Koning, BJC Middelkoop

**Affiliations:** 1 Department of Public Health and Primary Care, Leiden University Medical Center, Leiden, the Netherlands; 2 Department of Youth HealthCare, Centrum Jeugd en Gezin (Center for Youth and Family), The Hague, the Netherlands

**Keywords:** Child, Preschool, Growth charts, Public health/standards, Reference values, Body height, Body weight

## Abstract

**Objective:**

Several authors have questioned the suitability of WHO Child Growth Standards (WHO-CGS) for all ethnic groups. The aim of this study was to identify potential misclassification of stunting, underweight and wasting in children of Surinamese Asian Indian, South Asian (Pakistan/India) and Dutch descent.

**Design:**

A series of routine cross-sectional measurements, collected 2012–2015. South Asian-specific normative growth references for weight-for-age and weight-for-length/height were constructed using the LMS method based on historic growth data of Surinamese Asian Indians born between 1974 and 1976. WHO-CGS and ethnic-specific references were applied to calculate z-scores and prevalence of stunting, underweight and wasting.

**Setting:**

Youth HealthCare, providing periodical preventive health check-ups.

**Participants:**

11 935 children aged 0–5 years.

**Results:**

Considerable deviations from WHO-CGS were found, with higher-than-expected stunting rates, especially in the first 6 months of life. Surinamese Asian Indian children showed stunting rates up to 16·0 % and high underweight and wasting over the whole age range (up to 7·2 and 6·7 %, respectively). Dutch children consistently had mean WHO-CGS z-scores 0·3–0·5 sd above the WHO baseline (>6 months). The application of ethnic-specific references showed low rates for all studied indicators, although South Asian children were taller and larger than their Surinamese Asian Indian counterparts.

**Conclusions:**

WHO-CGS misclassify a considerable proportion of children from all ethnic groups as stunted in the first 6 months of life. Underweight and wasting are considerably overestimated in Surinamese Asian Indian children. Ethnic-specific growth references are recommended for Surinamese Asian Indian and Dutch children. The considerable differences found between South Asian subpopulations requires further research.

Growth is an important indicator of a child’s health^(1)^. To optimally monitor growth during childhood, the WHO established universal Child Growth Standards (WHO-CGS) for children aged 0–5 years for length/height-for-age, weight-for-age, weight-for-length/height and BMI-for-age^(2,3)^ in 2006. These standards were derived from combined growth data of affluent populations from Brazil, Ghana, India, Norway, Oman and the USA, representing all continents. The populations were carefully selected for growth under ‘optimal conditions’. As the distribution of growth data were highly similar across the different ethnic groups between birth and 5 years, the growth standards are generally considered universal for this age range and represent how children ‘should grow’^(2,3)^; as such, it was implemented in over 125 countries^(4)^. To detect abnormal growth and/or nutritional problems, the WHO defined different indicators based on cut-off centiles: stunting (height-for-age < –2 sd), wasting (weight-for-height < –2 sd) and underweight (weight-for-age < –2 sd)^(5)^.

Several studies have raised doubts about the applicability of such normative child growth standards to all ethnic groups^(6–8)^. South Asia still has the highest rates of stunting (39 %), underweight (29 %) and wasting (15 %) of children under 5 in the world^(9)^, despite having lower poverty levels and more favourable socioeconomic conditions than Sub-Saharan Africa^(10,11)^ Even in a high-income country such as the Netherlands, implausibly high thinness rates were found in affluent children of South Asian descent based on WHO cut-off criteria; however, this was not the case when using ethnic-specific BMI criteria^(12,13)^. Consequently, the high prevalence found using WHO BMI criteria could be down to misclassification. As South Asians are characterised by a predisposition to a ‘thin-fat’ phenotype, essentially a muscle-thin but adipose body composition, from birth until adulthood^(14–16)^, ethnic-specific growth standards/references are likely to better estimate the nutritional status. However, normative weight-for-age and weight-for-height references are not yet available for this group.

With regard to height, Asian children are also among the shortest in the world^(17)^, while Dutch children are the tallest^(18)^. Due to these differences, Dutch growth curves for the children of Dutch descent are likely to be an insufficient representation of the growth of children of South Asian descent living in the Netherlands^(19)^; the universal WHO-CGS may not be applicable in this case either.

To our knowledge, the prevalence of stunting, wasting and underweight among South Asian children living in affluent countries has not yet been studied. Whether stunting, wasting and underweight rates in Dutch children aged 0–5 in the Netherlands based on ethnic-specific references differ from those derived from WHO-CGS is unknown. It is also unknown whether the largest population of South Asian descent in the Netherlands, which originates from the former Dutch colony Suriname, is similar in growth to children from parents born in India or Pakistan.

The main objective of this study was to identify potential misclassification of stunting, wasting and underweight based on WHO-CGS in 0–5-year-old children of Dutch descent and in two distinct populations of 0–5-year-olds of South Asian descent: one originating from the Dutch ex-colony Suriname and the other directly from South Asia (India and Pakistan).

## Methods

### Population

Most South Asians in the Netherlands are descendants of Asian Indians who migrated from India to Suriname, a former Dutch colony, between 1873 and 1916. When Suriname became independent from the Netherlands in 1975, many of these Surinamese Asian Indians migrated to the Netherlands, especially to larger cities. The total population of Asian Indians with Surinamese ancestry in the city of The Hague is estimated at around 35 000^(20)^. Almost 5000 originate directly from India and Pakistan^(21)^.

### Data collection

Youth HealthCare in the Netherlands routinely offers periodical health assessments to all children living in the Netherlands, of which at least ten are conducted from birth till the age of 5; the results are recorded. The current study was based on this routine data.

Firstly, growth data of a historic cohort of affluent Surinamese children of Asian Indian descent, which was previously used to construct normative ethnic-specific BMI-for-age curves^(22)^, were used in the current study to develop ethnic-specific weight-for-age and weight-for-length/height curves for children 0–5 years of age. As children from this cohort were born between 1974 and 1976 (and as such represent the period before the obesity epidemic) and had no known growth constraints, their growth data were intended to set the norm for a ‘normal’ weight-for-age and weight-for-length/height in children of Asian Indian descent. Data extraction and handling were previously published^(22)^.

The second dataset used for the current study was also based on a series of routine growth measurements in Youth HealthCare, but now from contemporary children. All length/height and weight data of children aged 0–5 years (0–60 months), measured between 1 January 2012 and 31 December 2015, were extracted from digital health records of the Youth HealthCare centre in the city of The Hague (the Netherlands). Additionally, demographic data, such as parental and child family names, date of birth, sex, parental country of birth and parental level of education, were obtained from the Youth HealthCare database. For this study, the level of education was divided into three subgroups: low (≤prevocational education), middle and high (≥undergraduate), according to parents with the highest level of education.

### Inclusion criteria

Children were classified as Surinamese Asian Indian if they met two criteria: both parents had to be born in either Suriname (first generation) or the Netherlands (second generation) and both parents needed to have a Surinamese Asian Indian family name, which was determined by matching the names with a list of almost 2500 (Surinamese) Asian Indian family names. To determine an Indian or Pakistani ethnicity, both parents had to be born in either India or Pakistan. Dutch children were included if both parents were listed as born in the Netherlands and they had a typical Dutch family name.

Similar to the data selection of WHO-CGS, only measurements of children who were born singleton at term (≥37 and <42 weeks of gestation) were included. The criterion of exclusive breastfeeding for at least 4 months followed by partial breastfeeding up to 12 months could not be fulfilled as this information was unavailable or incompletely recorded. Children <2500 g were not excluded as this is generally regarded as normal, considering the variation within a normal distribution.

### Anthropometric measurements, references and cut-offs

Length/height and/or weight were measured by trained Youth HealthCare physicians, nurses or medical assistants at each routine health assessment. Between birth and the age of 18 months, length was measured with a measuring board in recumbent position with head against the base of the board, fully stretched legs and soles against the end of the measuring board. The length was read to the nearest 0·1 cm. A calibrated baby scale was used to measure weight to the nearest 0·01 kg. In children >18 months, standing height was measured using a calibrated stadiometer that was read to the nearest 0·1 cm. A physician’s balance scale or calibrated mechanical weight scale was used to measure weight, rounding to the nearest 0·1 kg. All measurements were registered on paper health records or the digital record system of Youth HealthCare.

Several growth references were used to calculate length/height-for-age, weight-for-age and weight-for-length/height z-scores for all measurements of contemporary children (Table 1). Firstly, WHO-CGS from the WHO Multicenter Growth Reference Study^(2)^ was used as a universal reference to measurements of the three ethnic groups. Secondly, length/height-for-age z-scores were calculated using the (descriptive) Dutch and Surinamese Asian Indian references of the fifth national growth study (measured 2007–2010)^(18,19)^, of which the latter was also applied to measurements of children of South Asian descent (Pakistan/India). Finally, weight-for-age and weight-for-length/height z-scores for Dutch children were based on the third national growth study references (1978–1979)^(23)^, which are considered normative as the included children were not or minimally influenced by the obesity epidemic. The weight-for-age and weight-for-length/height references created in the current study are also intended as normative references and were applied to all measurements of children of Surinamese Asian Indian and South Asian descent in the current study.

**Table 1:** Sources of different ethnic-specific growth references

	Dutch	Surinamese Asian Indian	South Asian
Length/height-for-age	5th NGS, Dutch participants^(18)^	5th NGS, SAI participants^(19)^	5th NGS, SAI participants^(19)^
Weight-for-age	3rd NGS, Dutch participants^(23)^	74–76 study, SAI participants^(22)^	74–76 study, SAI participants^(22)^
Weight-for length/height	3rd NGS, Dutch participants^(23)^	74–76 study, SAI participants^(22)^	74–76 study, SAI participants^(22)^

NGS, National Growth Study; SAI, Surinamese Asian Indian; SA, South Asian.

Stunting, wasting and underweight were defined as length/height-for-age, weight-for-length/height and weight-for-age <–2 sd ^(5)^. When considering a normal (Gaussian) distribution, a prevalence of 2·3 % is to be expected.

### Statistical analyses

Based on the growth data from the Surinamese Asian Indian cohort between 1974 and 1976, ethnic-specific weight-for-age and weight-for-length/height references were calculated, separately for males and females, by employing the LMS method^(24)^ in R (v 3.3.2) with the package for Generalised Additive Models for Location, Scale and Shape (GAMLSS)^(25)^. This method results in a reference that can be described by three curves: skewness (L-curve), median (M-curve) and coefficient of variation (S-curve). The fit of the LMS model to the data and the necessary amount of smoothing of the curves were determined by assessing ‘worm plots’ (AGD package for R), which are essentially (diagnostic) tools for analysing the residuals^(26)^. To achieve a stable fit of the LMS model at 5 years of age, measurements of children aged 5–9·99 were also added to the dataset before fitting. To establish a proper fit in periods of rapid growth, age was cube root-transformed for the weight-for-age references, and for the weight-for-length/height references, the length/height value was log-transformed. After an optimal fit was achieved, the transformed values were scaled back to the original values.

Descriptive statistics of the data of contemporary children (2012–2015) were calculated using Chi-square (categorical variables) and ANOVA (continuous variables). Ethnic differences in z-scores of length/height-for-age, weight-for-age and weight-for- length/height were determined using a linear model within Generalised Estimating Equations (GEE), which was set to account for the repeated measures (autoregressive model; AR1). The specific z-scores based on either WHO-CGS or ethnic-specific references were the dependent variable, and ethnicity was the independent variable, while adjusting for sex and parental level of education. Within this procedure, Estimated Marginal Means (EMM) by age were calculated, which for this study yielded z-score means by age that were adjusted for other factors in the model, also taking the correlation between repeated measurements into account.

To determine the prevalence of stunting, wasting and underweight, dichotomised variables (stunting *v.* no stunting, wasting *v.* no wasting, underweight *v.* no underweight) were calculated including the standard error. Statistical analyses were performed with IBM SPSS Statistics v24.

## Results

First, ethnic-specific growth references for weight-for-age and weight-for-length/height were constructed based on the growth of a cohort of 954 Surinamese Asian Indian children (51·3 % boys) born between 1974 and 1976. For the construction of weight-for-age references, the total sample included 7342 measurements of weight, while for weight-for-length/height references, 4667 measurements of height and weight were included. The resulting growth references, including the LMS values, can be found in online Supplemental file S1.

The study of growth of contemporary children had a total of 59 495 measurements from 11 935 children (Table 2). Most children were of Dutch descent (85 %). Parents of the children of Surinamese Asian Indian descent had the lowest education level compared with parents of the children of Dutch and South Asian descent.

**Table 2:** Study population characteristics by ethnic group

	Dutch (*n* 50 918)		Surinamese Asian Indian (*n* 7482)		South Asian (*n* 1095)	
Number of children	10 089		1586		260	
	%		%		%	
Sex						
Boy	51·1		50·5		51·0	
Girl	48·9		49·5		49·0	
Gestational age in weeks*						
Mean		39·8		39·3		39·5
sd		2·1		2·1		2·1
Assessment age in months*						
1	13·3		12·1		12·2	
3	13·0		11·9		12·3	
6	12·4		11·6		12·0	
9	10·4		9·7		10·8	
11	10·2		10·0		9·1	
14	11·0		10·5		11·8	
18	9·3		9·6		9·8	
24	8·9		9·5		9·4	
36	6·7		8·0		6·9	
48	4·7		7·2		5·7	
Highest parental education level*						
Low	14·3		23·5		22·5	
Middle	20·3		39·4		13·3	
High	65·5		37·1		64·2	

* Difference between ethnic groups, *P* < 0·001.

### WHO Child Growth Standards

**Fig. 1:**
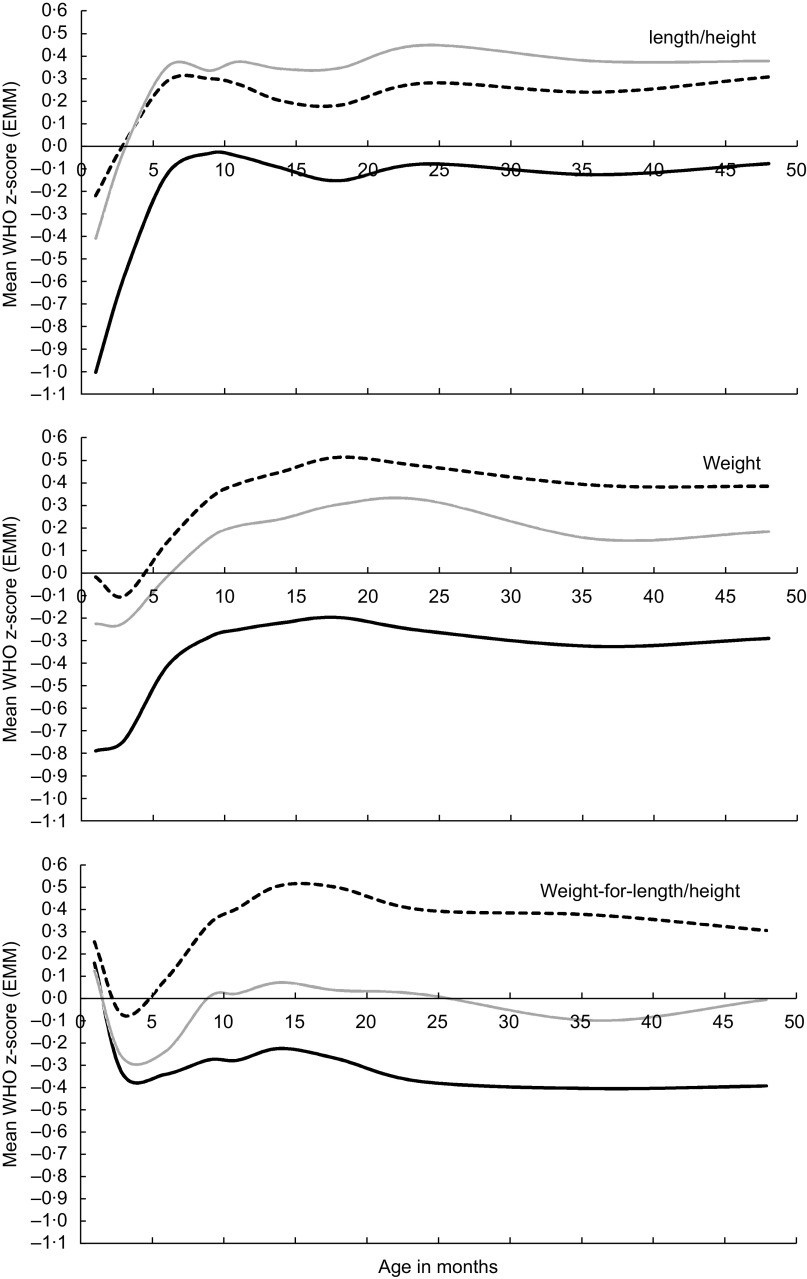
Application of WHO Child Growth Standards for length/height-for-age, weight-for-age and weight-for-length/height: (estimated marginal) mean z-scores by age in months and by ethnic group, adjusted for sex and parental education level (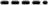, Dutch; 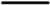, Surinamese Asian Indian; 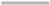, South Asian).

When applying WHO-CGS, a similar pattern for length/height was found in all three ethnic groups (Fig. 1). Compared with the (reference) WHO population, the length at 1 month of age was relatively short in all ethnic groups; this was especially true for Surinamese Asian Indian children. From that age onwards, length increased sharply to a stable trajectory from around 6 months of age in all ethnic groups to a mean z-score of –0·09 (95 % CI–0·14,–0·05) in children of Surinamese Asian Indian descent, and of 0·29 (95 % CI 0·27, 0·31) and 0·37 (95 % CI 0·22, 0·52) in children of Dutch and South Asian descent, respectively (data not shown).

Weight-for-age also showed a typical trajectory that was quite similar in the three ethnic groups, although the starting level differed. The initial level was relatively low, especially in the Surinamese Asian Indian group, which was followed by an increase between 3 and 12 months, after which a plateau was reached. Surinamese Asian Indian children had the lowest weight-for-age over the whole age range, consistently below the mean of the WHO standard. From around 10 months of age, children of Dutch and South Asian descent had, on average, a higher mean weight-for-age than WHO-CGS (*P* < 0·05).

In contrast to height-for-age and weight-for-age, weight-for-length/height started at a relatively high level in all ethnic groups, declining in the first 3 months, and then increasing to plateau at an age of 14 months. Children of Dutch descent had the highest weight-for-length/height z-scores, with values up to 0·50 (95 % CI 0·49, 0·53) at an age of 14 months, while children of Surinamese Asian Indian descent had the lowest z-scores, up to –0·40 (95 % CI –0·48, –0·33) at an age of 36 months, which are consistently below WHO-CGS over the whole age range.

At most ages, length/height-for-age, weight-for-age and weight-for-length/height z-scores were significantly higher in South Asian children than in children of Surinamese Asian Indian descent (data not shown).

When stunting, wasting and underweight criteria (<–2 sd) of the WHO reference (Table 3) were applied, a relatively high prevalence of stunting at 1 month of age was evident in all ethnic groups, which declined to the range of expected values at an age of 6 months. The highest level of stunting was found in children of Surinamese Asian Indian descent (16·0 %; 95 % CI 13·6, 18·4 %); simultaneously they had a relatively high underweight prevalence at 1 month of age (7·2 %; 95 % CI 5·5, 8·8 %), which decreased to near-normal levels at 18 months after which it increased again to higher-than-expected rates, up to 4·9 % (95 % CI 3·1, 6·6 %), at 36 months. Also, children of Surinamese Asian Indian descent had higher-than-expected wasting rates over almost the whole age range, up to 6·7 % (95 % CI 4·7, 8·7 %), at 36 months.

**Table 3:** Prevalence of stunting, wasting and underweight in Dutch, Surinamese Asian Indian and South Asian children by age in months, based on WHO and ethnic-specific criteria

	Stunting (length < –2 sd)						Underweight (weight < –2 sd)						Wasting (weight-for-length/height < –2 sd)					
	Dutch		Suriname Asian Indian		South Asian		Dutch		Suriname Asian Indian		South Asian		Dutch		Suriname Asian Indian		South Asian	
Age	%	se	%	se	%	se	%	se	%	se	%	se	%	se	%	se	%	se
WHO																		
1	4·4	0·25	16·0	1·23	5·3	1·94	2·0	0·17	7·2	0·86	3·0	1·49	1·3	0·14	1·2	0·37	3·0	1·49
3	2·4	0·19	7·3	0·87	4·5	1·79	1·8	0·16	7·1	0·86	2·2	1·28	2·5	0·19	4·2	0·67	3·7	1·64
6	1·0	0·13	3·2	0·60	0	0	0·8	0·11	5·4	0·77	1·5	1·07	1·3	0·14	3·1	0·59	2·3	1·30
9	1·2	0·15	2·1	0·53	2·6	1·49	0·7	0·12	4·2	0·75	0·9	0·87	0·8	0·12	4·2	0·75	3·5	1·72
11	1·2	0·15	1·9	0·50	1·0	0·98	0·6	0·10	3·5	0·68	1·0	0·98	0·5	0·09	3·0	0·62	2·9	1·68
14	1·3	0·15	3·3	0·63	1·6	1·10	0·4	0·08	3·8	0·68	0·8	0·78	0·2	0·06	2·5	0·56	0·8	0·78
18	1·2	0·16	3·0	0·64	2·8	1·59	0·3	0·08	2·7	0·61	0·9	0·93	0·3	0·07	2·6	0·59	0·9	0·93
24	0·8	0·13	3·0	0·64	2·0	1·38	0·3	0·08	4·6	0·78	2·9	1·68	0·3	0·08	5·5	0·85	3·9	1·93
36	0·9	0·16	3·2	0·72	0	0	0·4	0·11	4·9	0·88	0	0	0·2	0·07	6·7	1·02	1·3	1·27
48	0·7	0·17	2·1	0·61	1·6	1·61	0·4	0·13	3·5	0·80	0	0	0·3	0·12	5·4	0·98	1·6	1·61
Ethnic-specific																		
1	2·1	0·18	3·5	0·62	0·8	0·78	1·6	0·15	0·5	0·22	0·8	0·75	0·4	0·08	0	0	0·8	0·76
3	1·0	0·12	2·8	0·55	1·5	1·05	0·8	0·11	0·4	0·22	0	0	0·3	0·07	0·3	0·19	0	0
6	0·9	0·12	1·5	0·41	0	0	0·7	0·11	1·7	0·44	0	0	0·6	0·10	0·1	0·12	0	0
9	1·6	0·18	1·1	0·39	0·9	0·87	1·5	0·17	1·5	0·46	0·9	0·87	0·9	0·13	0·8	0·34	0	0
11	1·6	0·17	1·1	0·38	1·0	0·98	1·8	0·18	1·6	0·46	1·0	0·98	1·0	0·14	0·9	0·36	0	0
14	1·7	0·17	2·3	0·53	1·6	1·10	2·6	0·21	1·8	0·47	0·8	0·78	1·4	0·15	0·6	0·28	0	0
18	1·6	0·18	1·4	0·45	1·9	1·30	3·2	0·26	1·0	0·37	0·9	0·93	2·0	0·20	0·4	0·24	0	0
24	2·0	0·21	2·5	0·58	2·0	1·38	2·8	0·25	1·1	0·39	1·0	0·98	2·2	0·22	1·4	0·43	0	0
36	2·7	0·28	3·2	0·72	0	0	2·8	0·28	1·0	0·41	0	0	1·9	0·23	2·0	0·57	1·3	1·27
48	2·4	0·31	3·4	0·78	1·6	1·61	3·1	0·35	0·4	0·26	0	0	2·0	0·29	0·4	0·26	0	0

For all indicators, Dutch children had consistently lower rates at most ages than their Surinamese Asian Indian and South Asian counterparts. Wasting in South Asian children was quite high, up to an age of 9 months, after which the prevalence fluctuated around near-normal levels.

### Ethnic-specific growth references

**Fig. 2:**
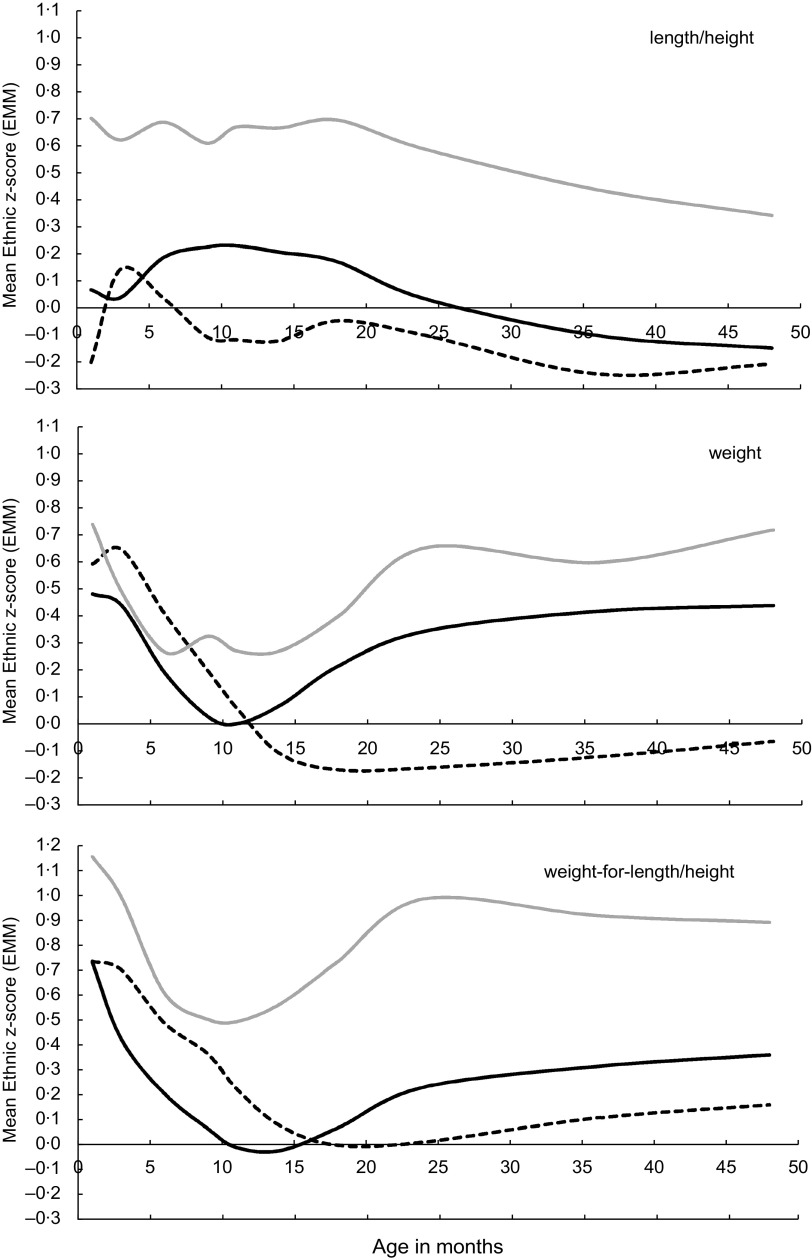
Application of ethnic-specific references for length/height-for-age, weight-for-age and weight-for-length/height: (estimated marginal) mean z-scores by age in months and by ethnic group, adjusted for sex and parental education level (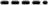, Dutch; 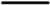, Surinamese Asian Indian; 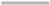, South Asian)

With ethnic-specific normative references applied, length/height-for-age showed largely similar trajectories for the three ethnic groups, with more or less stable levels up to 18 months, after which length/height-for-age declined (Fig. 2). Surinamese Asian Indian and South Asian children also had similarly shaped curves for weight-for-age and weight-for-length/height, although the mean z-scores of South Asian children were consistently higher than that of Surinamese Asian Indian children over almost the whole age range.

At 1 month after birth, the weight-for-length/height levels were high in all ethnic groups, even reaching up to +1·15 sd in South Asian children. Subsequently, the levels dropped quite steeply to the bottom at around 10 months of age in Surinamese Asian Indian and South Asian children, and at 18 months in children of Dutch descent. Then the levels rose in both South Asian populations, while the levels in Dutch children remained near the baseline of 0 sd.

For the three ethnic groups, stunting, wasting and underweight rates based on ethnic-specific criteria were not significantly lower and did not significantly differ from the 2·3 % level at most ages (Table 3). Rates of wasting and underweight were especially low in the South Asian group.

## Discussion

This study showed that in the first 6–12 months of life, the growth pattern of children of all three ethnic groups deviated from WHO-CGS, after which it plateaued at a relatively stable level. The rates of stunting based on WHO-CGS were relatively high in the first month after birth in Dutch (4·4 %), Surinamese Asian Indian (16·0 %) and South Asian (5·3 %) children, but declined to the expected level of 2·3 % at 6 months of age in children of Surinamese Asian Indian and South Asian descent. However, in Dutch children of the same age, stunting, wasting and underweight rates were considerably lower than expected. The prevalence of wasting and underweight in children of Surinamese Asian Indian descent was high at most ages, up to 6·7 and 7·2 %, respectively, while in children of South Asian descent, these remained around the 2·3 % level.

At all ages in the three ethnic groups, the ethnic-specific criteria for stunting, underweight and wasting led to the expected 2·3 % (or even lower rates than expected), which suggests that the WHO-CGS criteria are likely to misclassify many children as stunted, underweight or wasted. Especially length-for-age and weight-for-age in the first 6–12 months showed large contrasts between WHO-CGS and ethnic-specific references. Where low but increasing values were found using WHO-CGS, the ethnic-specific references showed relatively high but declining values.

In addition, the generally high trajectories of all growth parameters in Dutch children after 12 months of age relative to WHO-CGS, together with very low rates of stunting, wasting and underweight, render WHO-CGS less suitable for this group. Equally, the opposite pattern found in (affluent) children of Surinamese Asian Indian descent, generally low trajectories of growth parameters together with high rates of underweight and wasting over the whole age range, showed the unsuitability of WHO-CGS for children of Surinamese Asian Indian descent.

Besides the Netherlands, other high-income countries also experience the same problem, with quite low values of length and weight shortly after birth when using WHO-CGS, which increase to normal or above-normal levels in the first year^(27)^, as well as generally high trajectories of mean z-scores based on WHO-CGS together with lower rates of stunting, underweight and wasting than expected. As children in those countries are generally taller and heavier than those in the WHO-CGS population, the distribution shifts to the right relative to WHO-CGS. Therefore, the detection of growth problems at the lower end of the scale – for instance, Turner syndrome – is delayed, while at the higher end, an overestimation of potential health problems may occur^(8,28–30)^. At the same time, in children of shorter and thinner populations, such as Surinamese Asian Indian children in our study, wasting and underweight are likely to be overdetected^(12,13)^. For all the above reasons, WHO-CGS was considered unsuitable for use in the studied populations^(27,28,31)^.

### Explaining the results

The deviations of height and weight from WHO-CGS, consistently found in many European and other high-income countries, suggest a structural or systematic error in WHO-CGS. Selective dropout from the WHO Multicenter Growth Reference Study was suggested as a plausible explanation^(32)^, as the data of 50 % of all children enrolled in that study were not used due to non-compliance to the rules set out at the start of the study. For instance, all children had to be exclusively breastfed^(3)^. As breastfed infants generally have a more rapid weight gain in the first 3–4 months of life than formula-fed infants, followed by a slower body growth between 3 and 12 months^(30)^, the weight patterns seen in other populations relative to WHO-CGS suggest that only very-well-growing babies might have been included.

The large discrepancies found in the current study among children of Surinamese Asian Indian descent are a magnification of this problem of WHO-CGS, as these children, despite their affluence, are generally lighter than other ethnic groups^(13)^. The current study also shows that this finding is not restricted to infancy alone: weight-for-age and weight-for-length/height were considerably lower over the whole age range, 0–5 years, leading to an overestimation of underweight and wasting rates.

Furthermore, instead of low mean z-score levels for weight and weight-for-height in the first 6–12 months, the application of ethnic-specific references even showed relatively high mean z-score levels in children from all ethnic groups. The higher levels in Dutch children may be the result of a general increase of mean birth weight of term babies in the past 30 years^(33)^, but in Surinamese Asian Indian children, the mean birth weight was found to be stable from 1974 to 2009^(34)^. The currently found higher level at 1 month of age may suggest a right shift of the normal curve, with less children below –2 sd (underweight and wasting) as a consequence.

Compared with people of European descent at similar weight or BMI levels, South Asian infants, children and adults generally have a smaller lean body mass and a larger (abdominal) fat mass. This typical ‘thin-fat’ body composition is described in South Asians living in South Asia, as well as South Asians living in Suriname and other countries^(14,16,35–38)^. However, in the present study, South Asian children in the Netherlands who originated directly from India or Pakistan differed considerably from Surinamese Asian Indian children in all growth parameters, although the growth patterns seemed similar. The length/height-for-age was higher than that of Dutch children, while the weight-for-age was only slightly lower. The weight-for length/height was, on average, in-between Surinamese Asian Indian and Dutch children. In Pakistani babies in the UK, a similar growth pattern was found compared with white children^(39)^.

Previously, the length/height-for-age references and BMI distribution of Surinamese Asian Indian children aged 5–20 in the Netherlands were shown to be quite similar to those based on the growth of affluent Asian Indian children^(19,22)^. Nevertheless, the South Asian population aged 0–5 in the Netherlands seemingly grow differently. Therefore, the current South Asian-specific references in the Netherlands, which were based on the growth of a norm population of Surinamese Asian Indian children living in the Netherlands, may not be applicable to this specific group. Considering the high parental level of education of these children, it is more likely that the South Asian population in the city of The Hague is a highly selective sample of Pakistani and Asian Indian children, who may not be representative of Pakistani or Asian Indian children in general. In addition, it cannot be ruled out that the high z-score levels of weight-for-age and weight-for length/height may indicate that the population of Asian Indian and Pakistani children in the Netherlands are in fact overfat.

### Practical implications

From a clinical perspective, growth references should be able to detect, with a reasonably high sensitivity and specificity, children at risk of disease or premature death. A universally applicable reference, such as WHO-CGS, should do this well, irrespective of ethnic origin. Because of the potential misclassifications when using WHO-CGS, many studies raised doubts about its suitability to detect such at-risk children, and recommended the use of local (or ethnic-specific or country-specific) references instead of WHO-CGS^(6,32,40,41)^. As such references can likely reduce the risk of misclassification, these have the potential of elucidating true differences in growth and nutritional status between ethnic groups. However, as the creation of such references is not always feasible, the use of WHO-CGS is an alternative, the interpretation of which justifies a critical eye.

To be able to properly assess the growth of children of Dutch and Surinamese Asian Indian descent, we recommend using local, ethnic-specific references available in the Netherlands. The use of WHO-CGS may have potential adverse effects in Surinamese Asian Indian children, such as overfeeding, which may ultimately result in a larger (abdominal) fat mass. Ethnic-specific references for weight-for-age and weight-for-height are likely to better reflect the typical body composition of Surinamese Asian Indian children compared with WHO-CGS. For children who directly originated from Pakistan or India, further research on the applicability of ethnic-specific references is recommended, preferably in relation to body composition measures. The found differences between the subpopulations of the same ethnic group of ‘South Asians’ stress that heterogeneity within populations should be taken into account when interpreting results based on one reference.

### Strengths and limitations

Strengths of this study are the large sample size, reliability of data and stringent criteria for determining ethnicity, which restricted the inclusion to only children whose both parents met the criteria for ethnicity. Moreover, analyses were adjusted for individual socioeconomic status (highest level of education of parents). A limitation of the current study is that normative references based on the 1974–1976 cohort could not be corrected for the correlation of longitudinal measurements, which may have influenced the precision of the centiles. Also, formula-fed children could not be excluded due to this information not being available. Although ethnic differences in breastfeeding in the Netherlands are known^(42)^, most infants in the Netherlands are still breastfed during the first 6 months^(43)^. Therefore, the effects of formula feeding on the results are expected to be attenuated. Because the general prevalence of growth disorders and the use of growth-interfering medication is low and most of the involved children receive paediatric care instead of Youth HealthCare, the number of measurements from such children in our database are expected to be low. Therefore, we expect a negligible influence of it on the results of this study.

## Conclusion

The use of WHO-CGS misclassifies a considerable proportion of children from all ethnic groups as stunted in the first 6 months of life, while underweight and wasting are considerably overestimated in children of Surinamese Asian Indian descent. To properly assess the growth of children aged 0–5 in the Netherlands, ethnic-specific growth references are recommended for use in Dutch and Surinamese Asian Indian children. Further research into the suitability of normative South Asian references for other groups originating from the South Asian subcontinent is needed.

## Supplementary material

For supplementary material accompanying this paper visit https://doi.org/10.1017/S1368980019004464. click here to view supplementary material
